# Identification of High-Risk Multiple Myeloma With a Plasma Cell Leukemia-Like Transcriptomic Profile

**DOI:** 10.1200/JCO.21.01217

**Published:** 2022-03-31

**Authors:** Davine Hofste op Bruinink, Rowan Kuiper, Mark van Duin, Tom Cupedo, Vincent H.J. van der Velden, Remco Hoogenboezem, Bronno van der Holt, H. Berna Beverloo, Erik T. Valent, Michael Vermeulen, Francesca Gay, Annemiek Broijl, Hervé Avet-Loiseau, Nikhil C. Munshi, Pellegrino Musto, Philippe Moreau, Sonja Zweegman, Niels W.C.J. van de Donk, Pieter Sonneveld

**Affiliations:** ^1^ Department of Hematology, Erasmus MC Cancer Institute, Rotterdam, the Netherlands; ^2^ Department of Immunology, Erasmus University Medical Center, Rotterdam, the Netherlands; ^3^ SkylineDx, Rotterdam, the Netherlands; ^4^ HOVON Data Center, Department of Hematology, Erasmus MC Cancer Institute, Rotterdam, the Netherlands; ^5^ Department of Clinical Genetics, Erasmus University Medical Center, Rotterdam, the Netherlands; ^6^ Myeloma Unit, Division of Hematology, University of Torino, Azienda Ospedaliero-Universitaria Città della Salute e della Scienza di Torino, Torino, Italy; ^7^ Unité de Génomique du Myélome, IUC-Oncopole, Toulouse, France; ^8^ Dana-Farber Cancer Institute, Harvard Medical School, Boston, MA; ^9^ “Aldo Moro” University School of Medicine, Unit of Hematology and Stem Cell Transplantation, AOUC Policlinico, Bari, Italy; ^10^ Hematology Department, University Hospital Hôtel-Dieu, Nantes, France; ^11^ Department of Hematology, Amsterdam UMC, Vrije Universiteit Amsterdam, Cancer Center Amsterdam, Amsterdam, the Netherlands

## Abstract

Primary plasma cell leukemia (pPCL) is an aggressive subtype of multiple myeloma, which is distinguished from newly diagnosed multiple myeloma (NDMM) on the basis of the presence of ≥ 20% circulating tumor cells (CTCs). A molecular marker for pPCL is currently lacking, which could help identify NDMM patients with high-risk PCL-like disease, despite not having been recognized as such clinically.

**METHODS:**

A transcriptomic classifier for PCL-like disease was bioinformatically constructed and validated by leveraging information on baseline CTC levels, tumor burden, and tumor transcriptomics from 154 patients with NDMM included in the Cassiopeia or HO143 trials and 29 patients with pPCL from the EMN12/HO129 trial. Its prognostic value was assessed in an independent cohort of 2,139 patients with NDMM from the HOVON-65/GMMG-HD4, HOVON-87/NMSG-18, EMN02/HO95, MRC-IX, Total Therapy 2, Total Therapy 3, and MMRF CoMMpass studies.

**RESULTS:**

High CTC levels were associated with the expression of 1,700 genes, independent of tumor burden (false discovery rate < 0.05). Of these, 54 genes were selected by leave-one-out cross-validation to construct a transcriptomic classifier representing PCL-like disease. This not only demonstrated a sensitivity of 93% to identify pPCL in the validation cohort but also classified 10% of NDMM tumors as PCL-like. PCL-like MM transcriptionally and cytogenetically resembled pPCL, but presented with significantly lower CTC levels and tumor burden. Multivariate analyses in NDMM confirmed the significant prognostic value of PCL-like status in the context of Revised International Staging System stage, age, and treatment, regarding both progression-free (hazard ratio, 1.64; 95% CI, 1.30 to 2.07) and overall survival (hazard ratio, 1.89; 95% CI, 1.42 to 2.50).

**CONCLUSION:**

pPCL was identified on the basis of a specific tumor transcriptome, which was also present in patients with high-risk NDMM, despite not being clinically leukemic. Incorporating PCL-like status into current risk models in NDMM may improve prognostic accuracy.

## INTRODUCTION

For over a century, the level of circulating tumor cells (CTCs) has been assessed in multiple myeloma (MM) to identify aggressive disease.^1^ Although MM is characterized by an intramedullary outgrowth of malignant plasma cells, the degree of hematogenous tumor cell dissemination is highly variable between patients. At diagnosis, CTCs are routinely quantified in peripheral blood by morphology and can be detected in the majority of patients with MM if flow cytometry is used.^2^ However, in only 2% of patients, these levels are ≥ 20% or ≥ 2 × 10^9^/L, which is pathognomonic for primary plasma cell leukemia (pPCL).^3,4^ Symptomatic MM patients with lower CTC levels at diagnosis are classified as newly diagnosed MM (NDMM), but these patients may still develop secondary PCL (sPCL) after treatment.^5^

**CONTEXT**

**Key Objective**
Primary plasma cell leukemia (pPCL) is clinically differentiated from newly diagnosed multiple myeloma (NDMM) on the basis of the presence of ≥ 20% circulating tumor cells. It remains unknown if certain NDMM tumors molecularly resemble pPCL and if this has any prognostic relevance. The aim of this study was to construct a transcriptional classifier for PCL-like disease and to evaluate its prognostic value in the context of conventional high-risk markers in NDMM.
**Knowledge Generated**
The current study shows that pPCL can not only be identified clinically but also molecularly. Moreover, it demonstrates that a subgroup of patients with NDMM have a similar tumor transcriptome to pPCL: PCL-like MM. PCL-like status was significantly associated with an inferior progression-free and overall survival in NDMM.
**Relevance (*S. Lentzsch*)**
This study identifies a novel group of high-risk NDMM that transcriptionally resembled pPCL despite not being clinically leukemic. Determination of PCL-like status helps to identify high-risk patients requiring intensified treatment.*
  *Relevance section written by *JCO* Associate Editor Suzanne Lentzsch, MD, PhD.

Clinically, pPCL is considered a high-risk disease entity within MM.^6^ Patients commonly present with a large tumor burden and extensive morbidity, show poor response to standard treatment, and have a dismal overall survival (OS).^5,7-9^ Yet, several reports have suggested that certain patients with NDMM experience an equally aggressive disease course to patients with pPCL, without having CTC levels ≥ 20%.^10-12^ The International Myeloma Working Group has therefore challenged the current diagnostic criteria for pPCL, which has prompted ongoing research efforts to identify these PCL-like patients in alternative ways.^8^

Disease aggressiveness in pPCL is considered to be reflected by the presence of significantly higher CTC levels than that in NDMM. Although this was previously hypothesized to be the result of a spillover from a large intramedullary tumor, evidence is accumulating that altered molecular features involved in cell adhesion, evasion of apoptosis, migration, bone marrow (BM) independence, and RNA metabolism are associated with this phenotype.^13-22^ Still, molecular determinants of PCL-like disease remain poorly understood, with conventional high-risk markers in NDMM (ie, t(4;14), t(14;16) and deletion of chromosome 17p13 (del17p13)) only being detectable in a subset of pPCL tumors.^23-29^

We therefore hypothesized that by molecularly classifying PCL-like disease, a novel high-risk biology could be unveiled that may already be detectable in patients with NDMM, despite not being clinically leukemic.

## METHODS

### Study Design

This study was conducted in two phases: (1) transcriptomic classifier construction for PCL-like disease, and (2) assessment of its prognostic significance in NDMM. All human investigations in this study were performed after approval by medical ethical committees. Patient samples were obtained with written informed consent, in accordance with the Declaration of Helsinki.

### Patient Selection

**FIG 1.**
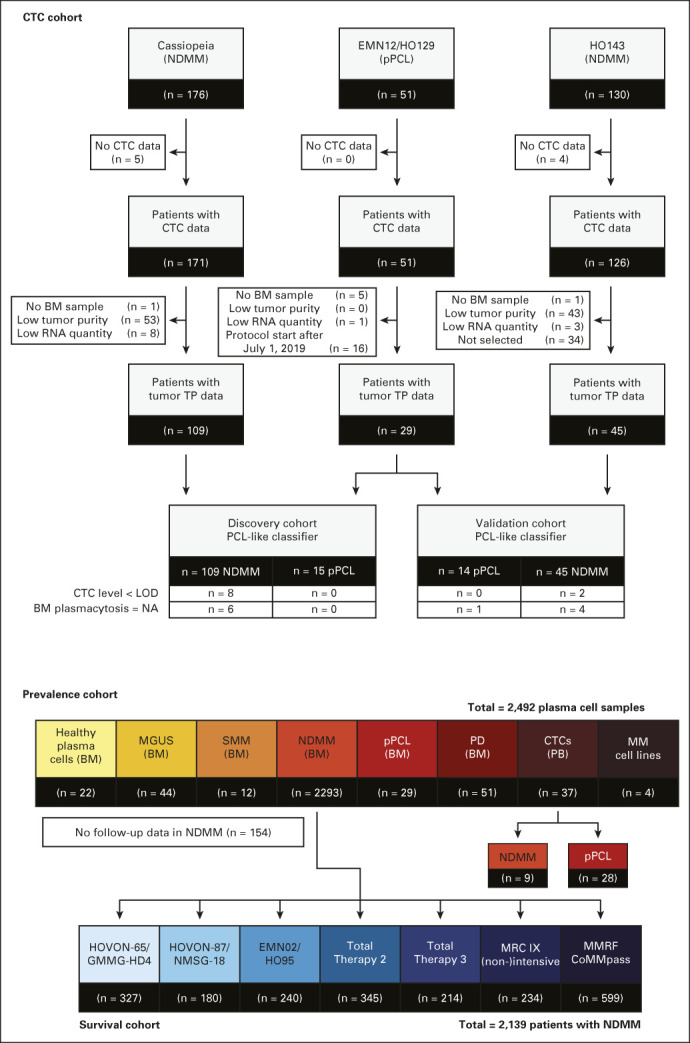
Study design. CONSORT diagram illustrating an overview of included patients in the study. CTC level, tumor transcriptomic, and tumor burden data from the discovery/validation cohort were used to construct and validate a molecular classifier for PCL-like disease. Transcriptomic profiling and follow-up data were leveraged to determine the prevalence of PCL-like disease in a wide range of plasma cell samples (prevalence cohort) and test its prognostic value in NDMM (survival cohort). BM, bone marrow; CTC, circulating tumor cell; LOD, limit of detection; MGUS, monoclonal gammopathy of undetermined significance; NA, not available; NDMM, newly diagnosed multiple myeloma; PB, peripheral blood; PD, progressive disease; pPCL, primary plasma cell leukemia; SMM, smoldering multiple myeloma; TP, transcriptomic profiling.

For classifier construction, patients with NDMM enrolled in the Cassiopeia (NCT02541383) or HO143 trials (EudraCT 2016-002600-90) and patients with pPCL from the EMN12/HO129 trial (EudraCT 2013-005157-75) were selected (CTC cohort; Fig 1).^30-32^ Patients with tumor transcriptomic profiles were divided into a discovery (n = 124) and validation cohort (n = 59).

PCL-like status prevalence per disease stage was assessed in 2,492 plasma cell samples (prevalence cohort). The prognostic value of PCL-like status was determined in a subset of 2,139 patients with NDMM from the prevalence cohort (survival cohort) with tumor transcriptomic, follow-up data and known age from the HOVON-65/GMMG-HD4 (EudraCT 2004-000944-26), HOVON-87/NMSG-18 (EudraCT 2007-004007-34), EMN02/HO95 (EudraCT 2009-017903-28), MRC-IX (ISRCTN68454111), Total Therapy 2 (NCT00083551), Total Therapy 3 (A: NCT00081939 and B: NCT00572169), and MMRF CoMMpass (NCT01454297) studies (Fig 1 and Data Supplement [online only, Supplementary Tables 1 and 2]).^33-39^

### CTC Level Quantification

Baseline CTC levels were determined by Next-Generation Flow (NGF; EuroFlow) in 297 patients with NDMM and by morphological assessment in 51 patients with pPCL.^40,41^

### Tumor Cell Transcriptomics

Transcriptomic profiles were generated from CD138-enriched BM tumor cells, using microarray or RNA Seq protocols. For technical validation, PCL-like scores were generated with both protocols in a subset of 123 samples.

### Classifier Construction

First, a linear regression model was used to rank genes on the basis of their association with high CTC levels, independent of tumor burden, defined as BM plasmacytosis. Second, the optimal number of genes to distinguish NDMM from pPCL was determined with a leave-one-out cross-validation analysis. Third, a cutoff was chosen for PCL-like disease by selecting the minimal PCL-like score to detect all pPCL tumors in the discovery cohort.

Classifier performance was tested in the validation cohort. Predicted CTC levels were calculated by fitting a linear model with both tumor burden data and PCL-like scores. The contribution of each term to the variance in observed CTC levels was determined by analysis of variance.

### Additional Molecular Testing

High-risk fluorescence in situ hybridization (FISH) was defined as the presence of del17p13, t(4;14), and/or t(14;16).^42^ Single sample gene set enrichment analysis (ssGSEA) scores and SKY92 high-risk, UAMS70 high-risk, and double-hit status were calculated as previously described.^43-47^

### Group Comparisons

Groups were compared using the two-sided Wilcoxon signed-rank or Wilcoxon rank-sum test for paired or unpaired continuous scenarios, respectively. The Fisher's exact test was used for categorical variables. Associations between continuous variables were tested by linear regression. *P* values were corrected for multiple testing according to the Benjamini-Hochberg procedure. *P* values and false discovery rates (FDRs) < .05 were considered statistically significant.

### Survival Analysis

For progression-free survival (PFS), an event was defined as either progressive disease (PD) or death from any cause. For OS, an event was defined as death from any cause.

Survival analysis was performed in R using the survival package (version 3.2.3), with the log-rank test to compare survival between groups.^48^ Meta-analysis was performed with the meta package (version 4.15.1), using a random effects model.^49^

Hazard ratios (HRs) were estimated from a Cox proportional hazards model stratified by the study cohort and including PCL-like status with age ≤ 65 years as covariates, in combination with Revised International Staging System (R-ISS) stage, ISS stage, high-risk FISH, SKY92 high-risk status, UAMS70 high-risk status, or high-risk cytogenetic aberrations.^42,50,51^ Two-sided *P* values < .05 were considered statistically significant for survival analyses.

Detailed information of all used methods is given in the Data Supplement (Supplementary Methods).

## RESULTS

### Baseline Characteristics of pPCL Versus NDMM

To investigate clinical and molecular determinants of PCL-like disease, baseline patient and tumor characteristics were collected for 297 NDMM and 51 pPCL patients with available CTC level data (CTC cohort; Fig 1 and Table 1). NGF was performed to quantify CTCs in patients with NDMM, which could be detected in 257 of 297 (87%) patients (range, 0.00028%-36%), with 40 of 40 (100%) CTC-negative assays reaching a limit of detection < 10^–5^ (Data Supplement, Supplementary Fig 1).

**TABLE 1.**
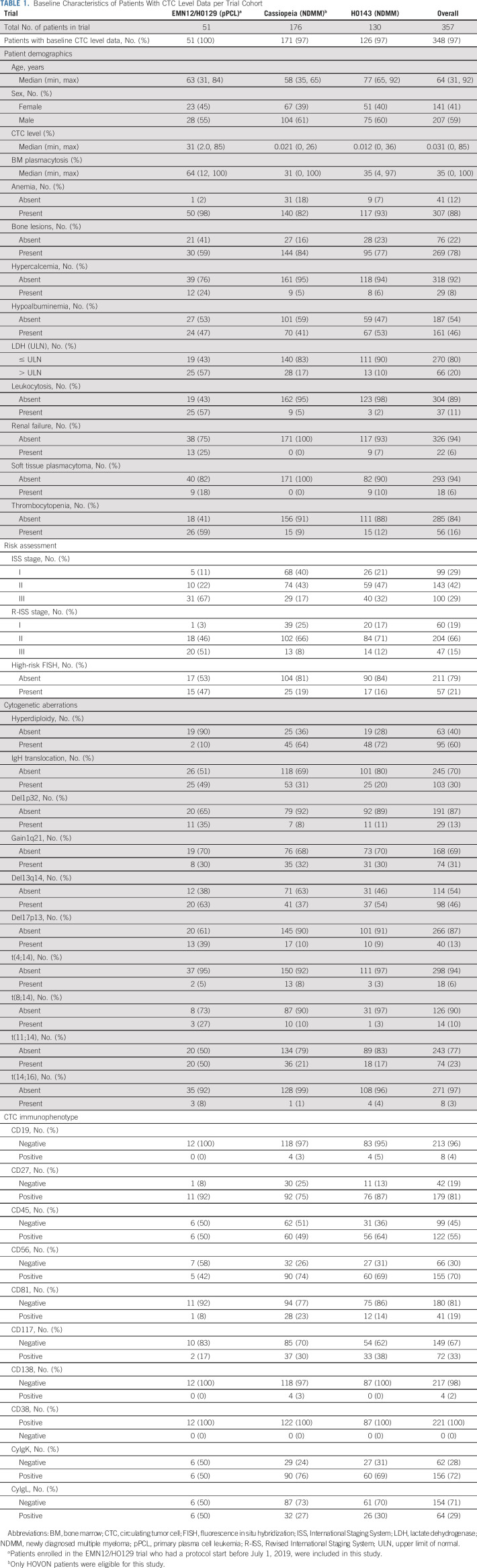
Baseline Characteristics of Patients With CTC Level Data per Trial Cohort

**FIG 2.**
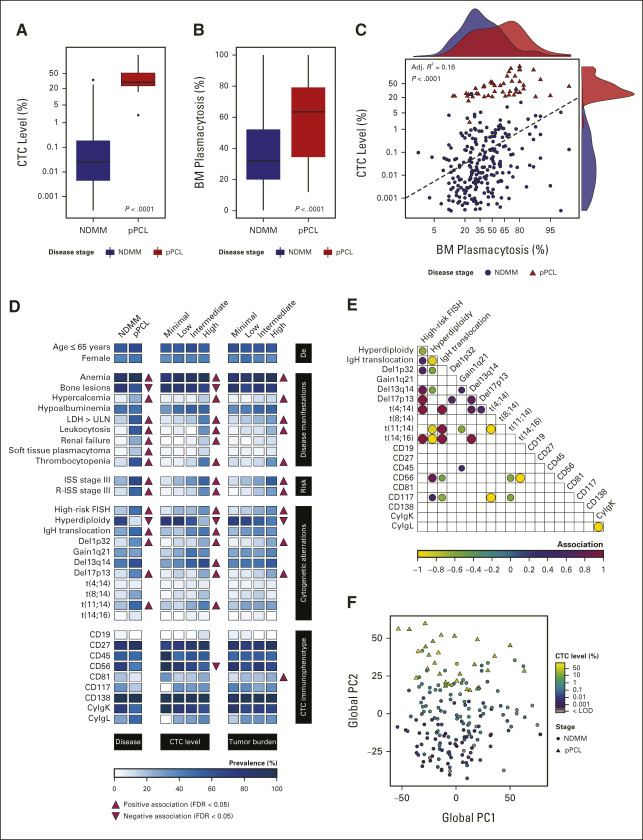
Clinical and molecular determinants of pPCL. (A) Boxplot showing CTC levels in pPCL (n = 51) and NDMM patients with detectable CTC levels (n = 257) from the CTC cohort. Data are shown on a log odds scale. The bold lines in the boxplots correspond to the median CTC level per disease stage, and the lower and upper hinges to the first and third quartiles. The whiskers extend to 1.5 times the interquartile range at most; data points beyond this level are depicted as outliers, represented by black dots. (B) Boxplot showing baseline tumor burden data between patients with pPCL (n = 50) and NDMM (n = 271) from the CTC cohort. (C) Combined scatter and density plot of tumor burden and CTC level data in NDMM patients with detectable CTC levels (n = 235) and pPCL (n = 50) from the CTC cohort. The dashed line represents the fitted linear model of the association between CTC level and tumor burden data, with the corresponding adjusted correlation coefficient and *P* value indicated in the upper left corner. Data are shown on a log odds scale. (D) Clinical, cytogenetic, and immunophenotypic baseline characteristics of patients with pPCL (n = 51) and NDMM (n = 297) from the CTC cohort. (E) Correlation plot demonstrating the co-occurrence of cytogenetic and immunophenotypic aberrations in NDMM (n = 297) and pPCL (n = 51) tumor samples from the CTC cohort. The size of the bubbles corresponds to the significance of the association, whereas the color of the bubbles reflects the scaled odds ratio as determined with the Fisher's exact test, followed by a sigmoid transformation. Only associations with an FDR < 0.05 are shown. (F) Global principal component analysis plot of all available transcriptomic profiles of pPCL (n = 29) and NDMM (n = 154) BM tumor samples from the CTC cohort, using all 12,928 expressed genes as input. CTC, circulating tumor cell; BM, bone marrow; De, demographics; FDR, false discovery rate; FISH, fluorescent in situ hybridization; ISS, International Staging System; LDH, lactate dehydrogenase; LOD, limit of detection; NA, not available; NDMM, newly diagnosed multiple myeloma; PC1, principal component 1; PC2, principal component 2; PCA, principal component analysis; pPCL, primary plasma cell leukemia; R-ISS, Revised International Staging System; ULN, upper limit of normal.

Both baseline CTC levels (median, 31% *v* 0.016%, *P* < .0001) and tumor burden as reflected by BM plasmacytosis (median, 64% *v* 32%, *P* < .0001) were higher in patients with pPCL than in patients with NDMM (Figs 2A and 2B). Tumor burden and CTC levels showed a positive, yet weak association (Adj. *R*^2^, 0.16, *P* < .0001), with all pPCL samples having higher CTC levels than expected on the basis of their tumor burden (Fig 2C).

Patients with pPCL presented with significantly higher morbidity than patients with NDMM, including more hypercalcemia (24% *v* 6%), renal failure (25% *v* 3%), and soft tissue plasmacytomas (18% *v* 3%), yet a lower occurrence of bone lesions (59% *v* 81%; FDR < 0.05; Fig 2D). Moreover, high-risk FISH status (47% *v* 18%) and the presence of an IgH translocation (49% *v* 26%), del1p32 (35% *v* 10%), del17p13 (39% *v* 10%), and t(11;14) (50% *v* 19%) were all more frequently detected in pPCL than in NDMM, whereas hyperdiploidy was less observed in pPCL (10% *v* 68%; FDR < 0.05). Of note, 15 of 16 (94%) PCL-like features identified in this analysis were also significantly associated with the CTC level (FDR < 0.05), whereas 11 of 16 (69%) PCL-like features were also significantly associated with tumor burden. Many molecular aberrations co-occurred (Fig 2E).

### A Transcriptomic Profile Representing PCL-like Disease

To enable a more comprehensive screening of tumor cell aberrations that are associated with PCL-like disease, transcriptomic profiling was performed for BM tumor cells in a subgroup of 154 patients with NDMM and 29 patients with pPCL from the CTC cohort (Fig 1 and Data Supplement [Supplementary Tables 1 and 3]). In a global principal component analysis (PCA) using all 12,928 genes that were expressed in these 183 samples, pPCL samples clustered together. Yet, a subgroup of NDMM samples had a highly similar transcriptomic profile to pPCL samples and these generally had CTC levels that were above average for NDMM (Fig 2F).

To identify essential genes defining this PCL-like transcriptome, a linear model was applied, in which the CTC level was used as a surrogate marker for PCL likeness, rather than comparing pPCL with NDMM samples in a dichotomous model. After correction for tumor burden, 1,700 genes were identified, which had a significant association with the CTC level in the discovery cohort (FDR < 0.05). These genes were among others involved in cell adhesion (eg, *NCAM1*, *ITGA6*, and *SDC1*), tumor suppression (eg, *PTEN*, *TUSC2*, and *TAGLN2*), proliferation (eg, *MKI67*, *MCM2*, and *CENPM*), RNA splicing (eg, *SRSF10*, *SF3A2*, and *PUF60*), cell migration (eg, *ROCK1*, *DOCK11*, and *DLC1*), and DNA damage control (eg, *CHEK1*, *DCLRE1C*, and *SLFN11*; Data Supplement [Supplementary Table 4]).^52-66^

**FIG 3.**
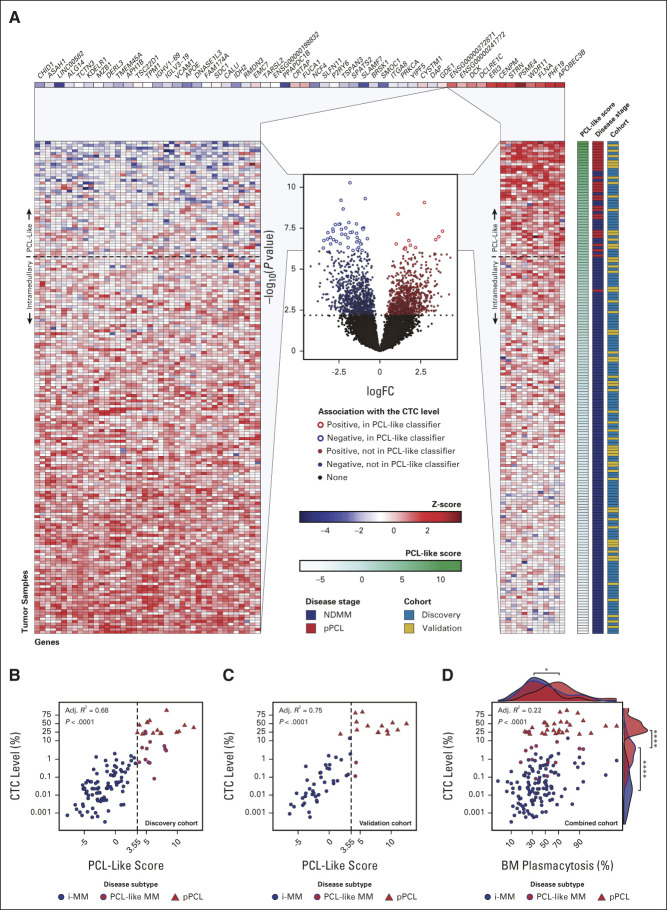
Construction and validation of a molecular classifier for PCL-like disease. (A) Volcano plot showing all 12,928 expressed genes, of which, the association with high CTC levels was tested in the discovery cohort (n = 95 NDMM and n = 15 pPCL patients). The log fold change corresponds to the change in gene expression per log odds unit increase in the CTC level, independent of tumor burden. There were 1,700 genes that showed a significant association, which are depicted in color (FDR < 0.05). The open circles represent the 54 most significant genes that have been selected for the PCL-like classifier. Their corresponding normalized expression values are shown in the heatmap for all available pPCL (n = 29) and NDMM (n = 154) BM tumor transcriptomes in the discovery/validation cohort. Gene symbols are displayed according to the HUGO Gene Nomenclature that corresponds to Ensembl release 74. Genes without a corresponding gene symbol in Ensembl release 74 are indicated with their Ensembl gene ID. (B) Scatter plot showing the association between the PCL-like score and the CTC level in the discovery cohort (n = 116 patients). The dashed line represents the lowest PCL-like score of pPCL samples in the discovery cohort (3.55), which is the threshold for the PCL-like classifier. NDMM samples with a PCL-like score ≥ 3.55 are classified as PCL-like MM; NDMM samples with a PCL-like score < 3.55 are classified as i-MM. The CTC level is displayed on a log odds scale. (C) Scatter plot showing the association between the PCL-like score and the CTC level in the validation cohort (n = 57 patients). The dashed line represents the threshold of the PCL-like classifier above which samples are classified as PCL-like. The CTC level is displayed on a log odds scale. (D) Combined scatter and density plots of tumor burden, CTC level, and disease subtype data for all patients from the discovery/validation cohort with available data (n = 121 patients with i-MM, n = 13 patients with PCL-like MM, and n = 28 patients with pPCL). The adjusted correlation coefficient and *P* value represent the association between BM plasmacytosis and the CTC level. In the density plots, PCL-like MM is compared with i-MM and pPCL, respectively. Only significant differences are shown. *P < .05; **P < .01; ***P < .001; and ****: *P* < .0001. BM, bone marrow; CTC, circulating tumor cell; i-MM, intramedullary multiple myeloma; logFC, log fold change; NDMM, newly diagnosed multiple myeloma; PCL-like MM, plasma cell leukemia-like multiple myeloma; pPCL, primary plasma cell leukemia.

By using the composite information of a selection of 54 of 1,700 genes, a score was constructed with which pPCL could be best distinguished from NDMM samples: the PCL-like score (Fig 3A, Table 2, and Data Supplement [Supplementary Fig 2 and Supplementary Table 5]). This score was independent of the platform that was used to generate it (microarray *v* RNA Seq), as evidenced by a high interplatform correlation of PCL-like scores in 123 paired samples (Adj.*R*^2^, 0.94; *P* < .0001; Data Supplement [Supplementary Fig 3 and Supplementary Tables 1 and 3]). In the validation cohort, 60% of the variance in CTC levels could be predicted by the PCL-like score and 6% by tumor burden, with observed CTC levels strongly correlating with the predicted CTC levels (Adj.*R*^2^, 0.79; *P* < .0001; Data Supplement [Supplementary Fig 4]).

**TABLE 2.**
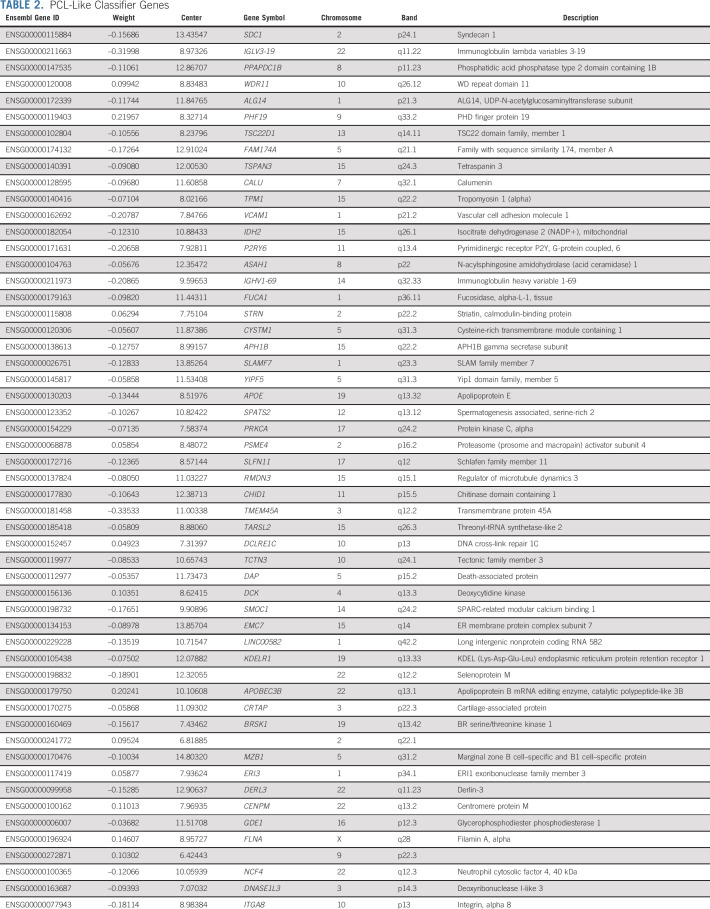
PCL-Like Classifier Genes

### Identification of PCL-like MM Tumors

Since the PCL-like score is a reflection of PCL-like disease, we hypothesized that this information could be leveraged to identify NDMM tumors with a similar transcriptome to pPCL tumors. To this end, a threshold for the PCL-like classifier was set (Fig 3B). With this threshold, 13 of 14 (93%) pPCL tumors in the validation cohort were correctly classified as PCL-like (Fig 3C). Of note, a subgroup of NDMM tumors was also classified as PCL-like on the basis of this threshold, despite presenting with CTC levels as low as 0.083%: PCL-like MM (Figs 3B and 3C). PCL-like MM had both lower CTC levels (median, 3.0% *v* 35%; *P* < .0001) and a lower tumor burden (median, 36% *v* 71%; *P* = .045) than pPCL (Fig 3D). Patients with NDMM who had a PCL-like score below 3.55 were referred to as intramedullary MM (i-MM).

**FIG 4.**
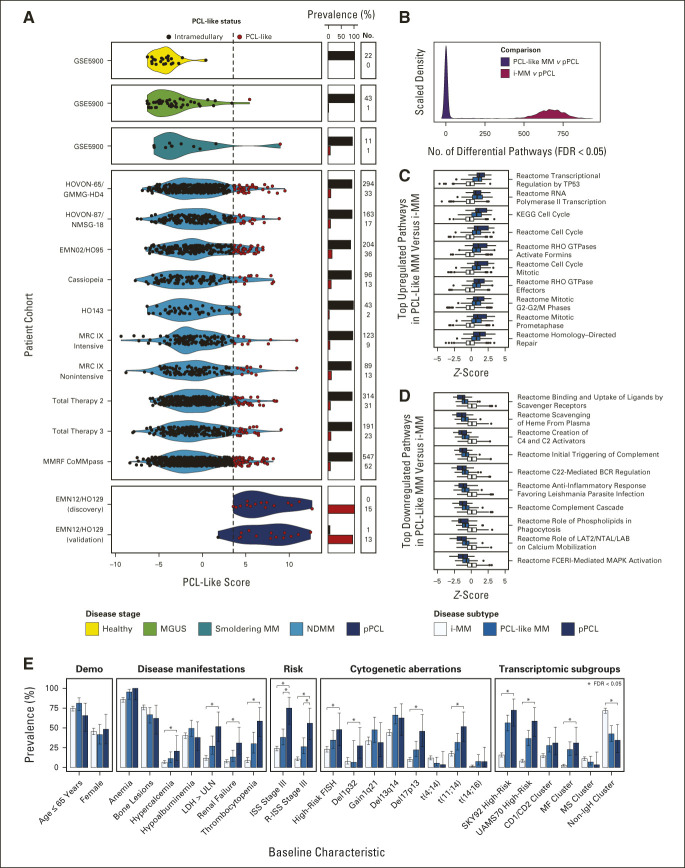
Clinical and molecular determinants of PCL-like MM. (A) Violin plot of PCL-like scores from healthy plasma cell, MGUS, SMM, NDMM, and pPCL BM tumor samples from the prevalence cohort (n = 2,400 patients), comprising 11 different data sets. Of note, certain patients with NDMM show extreme PCL-like scores, raising the possibility that these were patients with pPCL who were inadvertently included in NDMM trials. (B) Density plot showing the number of differentially expressed ssGSEA pathways (FDR < 0.05) per comparison between PCL-like versus pPCL and i-MM versus pPCL samples from the prevalence cohort (n = 757 i-MM, n = 99 PCL-like MM, and n = 29 pPCL samples). ssGSEA scores of 1,788 pathways were compared between 29 pPCL and a random sample of 29 i-MM or PCL-like samples, which was performed 1,000 times. (C) Box plots of 10 pathways that were most significantly upregulated in a subset of PCL-like MM (n = 99) versus i-MM samples (n = 757) from the prevalence cohort with a logFC > 0.75 (FDR < 0.05), displayed per disease subtype. The bold lines in the boxplots correspond to the median normalized ssGSEA scores per disease subgroup, and the lower and upper hinges to the first and third quartiles. The whiskers extend to 1.5 times the interquartile range at most; data points beyond this level are depicted as outliers, as represented by black dots. (D) Box plots of 10 pathways that were most significantly downregulated in a subset of PCL-like MM (n = 99) versus i-MM samples (n = 757) from the prevalence cohort, with a logFC < –0.75 (FDR < 0.05), displayed per disease subtype. (E) Bar charts comparing baseline characteristics of PCL-like MM with pPCL and of i-MM with pPCL in a subset of patients from the prevalence cohort. Error bars represent the 95% CI of the observed prevalence per disease subgroup, as determined with the Wilcoxon score interval with continuity correction. BM, bone marrow; Demo, demographics; FDR, false discovery rate; FISH, fluorescence in situ hybridization; i-MM, intramedullary multiple myeloma; ISS, International Staging System; LDH, lactate dehydrogenase; logFC, log fold change; MAPK, mitogen-activated protein kinase; MGUS, monoclonal gammopathy of undetermined significance; MM, multiple myeloma; NDMM, newly diagnosed multiple myeloma; PCL-like MM, plasma cell leukemia-like multiple myeloma; pPCL, primary plasma cell leukemia; R-ISS, Revised International Staging System; SMM, smoldering multiple myeloma; ssGSEA, single sample gene set enrichment analysis; ULN, upper limit of normal.

In all 10 NDMM cohorts of the prevalence cohort, a PCL-like transcriptome was consistently identified, with a prevalence ranging from 2 of 45 (4%; HO143 cohort) to 36 of 240 (15%; EMN02/HO95 cohort). PCL-like transcriptomes were not detected in healthy plasma cell samples, in 1 of 44 (2%) monoclonal gammopathy of undetermined significance samples, and in 1 of 12 (8%) smoldering MM (SMM) samples (Fig 4A). Ten of 51 (20%) PD and 34 of 37 (92%) CTC samples were classified as PCL-like, as well as 4 of 4 (100%) MM cell lines (Data Supplement [Supplementary Fig 5A]). Dividing NDMM and pPCL samples into four subgroups on the basis of previously reported transcriptomic clusters showed an enrichment of PCL-like status in the MF (74 of 136, 54%) and CD1/CD2 (74 of 374, 20%) clusters (Data Supplement [Supplementary Figs 5B-C]). Of note, no change in the PCL-like score was observed between paired NDMM and PD samples (*P* = .8), whereas both SKY92 (*P* = .0001) and UAMS70 high-risk scores (*P* = .0001) had increased significantly at the time of PD (Data Supplement [Supplementary Fig 6]).

### Molecular and Clinical Determinants of PCL-Like MM

A comparison of ssGSEA scores between subgroups showed that pPCL and i-MM were highly distinct at the transcriptomic level, whereas PCL-like MM and pPCL were very similar (Fig 4B). A total of 1,160 pathways were differentially expressed between PCL-like MM and i-MM, which were among others involved in p53 signaling, Rho GTPase activity, mitosis, and binding and uptake of ligands (Figs 4C and 4D).

In addition, at the clinical and cytogenetic level, PCL-like MM was more similar to pPCL than i-MM. PCL-like MM only had a lower prevalence of R-ISS stage III (26% *v* 56%) and ISS stage III (38% *v* 75%) than pPCL, whereas i-MM differed from pPCL with respect to the presence of 14 of 25 investigated baseline characteristics, including del1p32 (8% *v* 27%), del17p13 (10% *v* 46%), and t(11;14) (17% *v* 52%; FDR < 0.05; Fig 4E).

Comparing all three transcriptomic classifiers head-to-head showed that the PCL-like classifier had the highest sensitivity to detect pPCL (93%). PCL-like scores were only weakly associated with both SKY92 (Adj.*R*^2^, 0.26; *P* < .0001) and UAMS70 scores (Adj.*R*^2^, 0.25; *P* < .0001; Figs 3C and 4A and Data Supplement [Supplementary Figs 7 and 8]). PCL-like status was associated with double-hit status (*P* = .007), but not with *TP53* mutational status (*P* = .34; Data Supplement [Supplementary Table 6]).

Of 37 patients with NDMM and pPCL, matched tumor samples from BM and peripheral blood were available. CTCs had a higher PCL-like score than matched BM tumor samples (median, 7.64 *v* 5.54, *P* = .0005). Of note, i-MM did not differ from pPCL regarding the PCL-like score of their CTCs (median, 7.10 *v* 7.42; *P* = .39), despite having a significantly lower PCL-like score of their BM tumors (median, 2.76 *v* 7.02; *P* = .0004; Data Supplement [Supplementary Fig 9]).

### PCL-like Status as an Independent Prognostic Marker in NDMM

The prognostic value of PCL-like status was evaluated in an independent cohort of 2,139 patients with NDMM, with a median follow-up time of 57.5 months and 214 of 2,139 (10%) patients being classified as PCL-like (Fig 1; Data Supplement [Supplementary Tables 1, 2, and 7]). Overall, PCL-like status conferred both a significantly worse PFS (HR, 1.85; 95% CI, 1.60 to 2.14) and OS (HR, 2.12; 95% CI, 1.78 to 2.51) in univariate meta-analyses (Data Supplement [Supplementary Fig 10]). This negative prognostic impact was largely irrespective of the received treatment, with the highest impact on PFS and OS observed in the Total Therapy 3 trial cohort, in which PCL-like status had a HR of 2.96 (95% CI, 1.56 to 5.61) and 3.33 (95% CI, 1.68 to 6.61), respectively.

**FIG 5.**
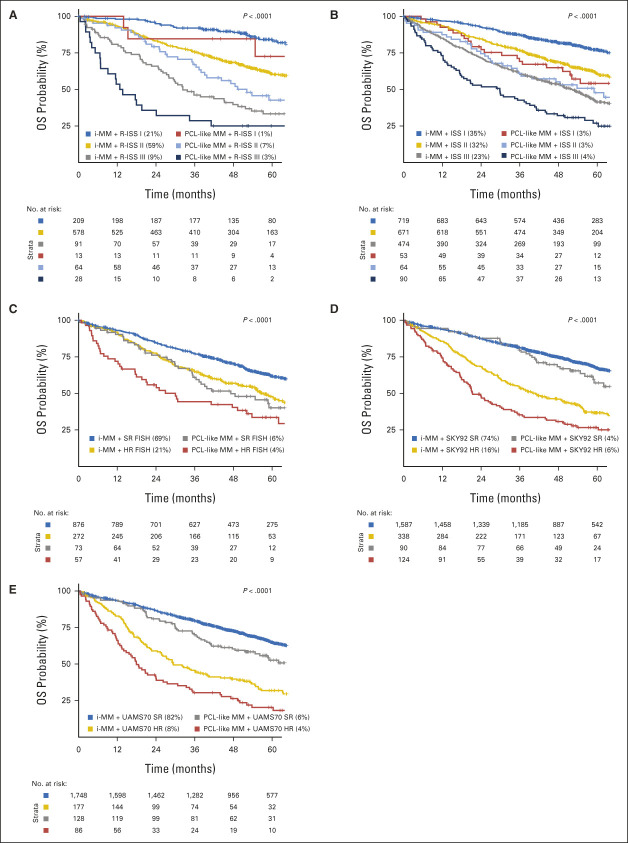
Kaplan-Meier plots of the association of PCL-like status with OS in combination with conventional prognostic factors in NDMM. *P* values represent the prognostic significance of the overall model. PCL-like status in combination with (A) R-ISS status, (B) ISS status, (C) high-risk FISH status, (D) SKY92 high-risk status, and (E) UAMS70 high-risk status. FISH, fluorescence in situ hybridization; i-MM, intramedullary multiple myeloma; ISS, International Staging System; HR, high-risk; NDMM, newly diagnosed multiple myeloma; OS, overall survival; PCL-like MM, plasma cell leukemia-like multiple myeloma; R-ISS, Revised International Staging System; SR, standard risk.

PCL-like status was significantly associated with both PFS and OS in the context of R-ISS stage, ISS stage, high-risk FISH, SKY92 high-risk status, UAMS70 high-risk status, t(4;14), t(14;16), del17p13, del1p32, and gain1q21 (Fig 5, Table 3, and Data Supplement [Supplementary Figs 11-13 and Supplementary Tables 7 and 8]). PCL-like MM patients with R-ISS stage III (28 of 983, 3%) had a median OS of only 13.2 months (95% CI, 6.8 to 41.1) versus not reached (95% CI, 87.8 to infinite) for i-MM patients with R-ISS stage I (209 of 983, 21%).

**TABLE 3.**
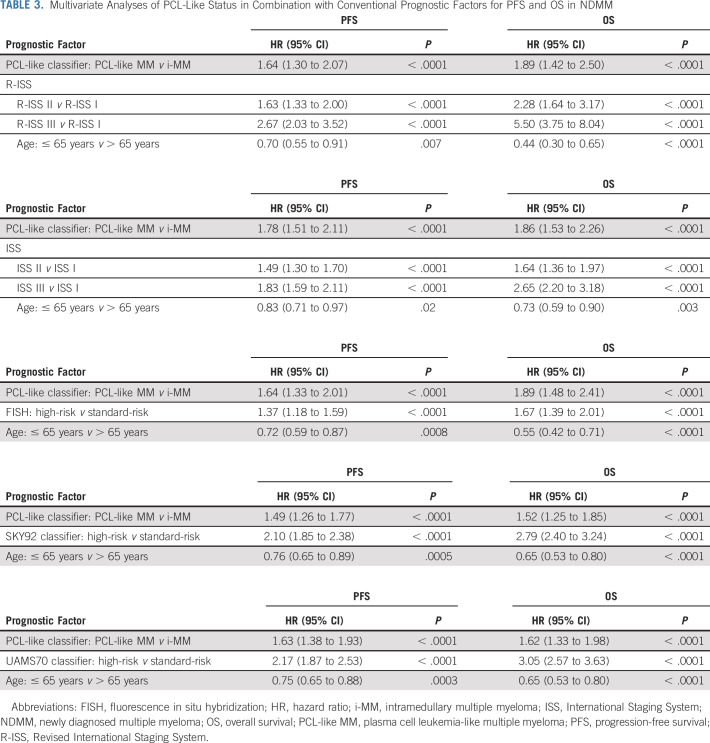
Multivariate Analyses of PCL-Like Status in Combination with Conventional Prognostic Factors for PFS and OS in NDMM

## DISCUSSION

In this study, a molecular classifier representing PCL-like disease was constructed and validated. On the basis of tumor transcriptomic data alone, this classifier not only identifies pPCL with a high sensitivity but also detects a PCL-like transcriptome in 10% of patients with NDMM, despite not meeting current diagnostic criteria for pPCL. PCL-like status had significant prognostic value in the context of conventional risk factors in NDMM and thereby represents a novel diagnostic tool to identify high-risk patients.

pPCL is an aggressive subtype of MM that in contrast to NDMM is characterized by high CTC levels. To the best of our knowledge, this is the first comprehensive study to demonstrate that pPCL can not only be classified at the clinical but also at the molecular level. This finding allowed for the identification of NDMM tumors with a PCL-like transcriptome, which demonstrated an enrichment of many characteristics that have been associated with pPCL previously, eg, increased proliferation, increased hypoxia, decreased expression of adhesion markers, assignment to the MF cluster, and the presence of t(11;14).^13,23,27,28,52,67-69^ A similar observation has recently been described with respect to the detection of NDMM-like mutational and copy number profiles in a subgroup of SMM, raising the question whether treatment decisions in MM should be driven by disease manifestations or rather by their molecular classification.^70-72^

Although PCL-like disease is associated with high CTC levels, it should be emphasized that a different group of patients with NDMM is identified with the PCL-like classifier than with clinically relevant CTC level thresholds (Data Supplement [Supplementary Fig 14]).^4,10-12,73-78^ Although 53% of PCL-like MM patients in our cohort had CTC levels ≥ 2%, which may represent underdiagnosed pPCL because of circadian fluctuations in CTC levels or atypical CTC morphology, a subgroup of NDMM patients with CTC levels as low as 0.083% also had BM tumor cells that transcriptionally resembled pPCL.^79,80^

We demonstrated that the lower CTC levels observed in PCL-like MM versus pPCL could be explained by a smaller tumor burden. Yet, about one third of CTC variance remains unexplained, which warrants further exploration. This information could be of particular relevance when studying the evolutionary biology of MM, as high CTC levels represent advanced disease.^81,82^ The observation that paired NDMM and PD samples in our study did not differ in terms of PCL-like score could suggest that PCL likeness is an inherent, rather than an acquired feature of MM tumors. In this regard, PCL-like status could be one of several conditions that need to be met for the development of sPCL, in combination with additional factors such as immune evasion or increased BM angiogenesis.^83,84^

Although PCL-like status is associated with both an inferior PFS and OS in univariate analyses, it is prognostically most valuable in combination with other risk models. Combining PCL-like status with R-ISS stage improved prognostic accuracy and enabled the identification of a subgroup of NDMM patients with exceptionally high-risk disease, as evidenced by a median OS of 13.2 months (95% CI, 6.8 to 41.1) for PCL-like MM patients with R-ISS stage III. Considering that most patients with pPCL in our study also presented with R-ISS stage III in combination with a PCL-like tumor transcriptome, it is remarkable to see that this OS in patients with NDMM agrees well with recently reported survival rates in pPCL.^85-93^ This suggests that a combination of R-ISS stage with PCL-like status may be used to identify borderline pPCL patients although it should be noted that many diagnostic laboratories still have limited experience with transcriptomic classifiers.

In the conducted meta-analyses, PCL-like status conferred high-risk disease irrespective of received treatment. Its association with an inferior PFS in 7 of 8 trial cohorts and an inferior OS in 6 of 8 trial cohorts underlines the unmet need to develop effective treatment strategies for PCL-like disease.^7,94-96^ Although preliminary reports suggest that novel agents including daratumumab and venetoclax are effective in pPCL, this study has identified additional potential therapeutic vulnerabilities in PCL-like tumors, which warrants further translational efforts.^97-99^ Examples include high expression of the nuclear export gene *XPO1* in PCL-like tumors, which is associated with sensitivity to selinexor, increased *PHF19* expression, and PRC2 pathway activity, which can be targeted by PRC2 inhibitors, and high levels of phosphorylating enzyme gene *DCK*, which has been found to confer a good response to nucleoside analogs.^100-102^

In conclusion, in this study a transcriptomic classifier for PCL-like disease was constructed and validated, which enables the identification of NDMM tumors with a similar molecular composition to pPCL and improves prognostic accuracy in NDMM in combination with conventional risk markers.

## Data Availability

Salmon TPM count data from the EMN02/HO95 and HO143 cohorts and CEL files from the EMN02/HO95, EMN12/HO129, and Cassiopeia cohorts are available on the GEO repository (https://www.ncbi.nlm.nih.gov/geo/), under accession codes GSE164847, GSE164830, GSE164706, GSE164703, and GSE164701, respectively (Data Supplement [Supplementary Table 1]).
